# Effect of Hydrogen Treatment on Photoluminescence and Morphology of InGaN Multiple Quantum Wells

**DOI:** 10.3390/nano12183114

**Published:** 2022-09-08

**Authors:** Yachen Wang, Feng Liang, Degang Zhao, Yuhao Ben, Jing Yang, Zongshun Liu, Ping Chen

**Affiliations:** 1 State Key Laboratory of Integrated Optoelectronics, Institute of Semiconductors, Chinese Academy of Sciences, Beijing 100083, China; 2 Collage of Materials Science and Opto-Electronic Technology, University of Chinese Academy of Sciences, Beijing 100049, China; 3 School of Electronic, Electrical and Communication Engineering, University of Chinese Academy of Sciences, Beijing 100049, China

**Keywords:** InGaN quantum well, hydrogen treatment, photoluminescence, surface morphology

## Abstract

In this paper, the photoluminescence (PL) properties and surface morphology of InGaN/GaN multiple quantum well (MQW) structures with the hydrogen (H_2_) heat treatment of InGaN are investigated to elucidate the effect of hydrogen on the structure and surface of the MQWs. The experimental results show that the H_2_ heat treatment on the as-grown MQWs may lead to the decomposition of InGaN and the formation of inhomogeneous In clusters. The atomic force microscope (AFM) study indicates that although the surface roughness of the uncapped samples increases after H_2_ treatment, the V-defects are suppressed. Moreover, the luminescence efficiency of the MQWs can be effectively improved by growing a GaN cap layer with an appropriate thickness on the top of the MQWs, which can reduce the effects of the H_2_ atmosphere and high temperature on the MQWs. In addition, a morphologic transformation from step bunching to shallow steps occurs and a much smoother surface can be obtained when a thicker cap layer is adopted.

## 1. Introduction

The wide luminous range (0.7–6.2 eV) of GaN-based multiple quantum well (MQW) heterostructures, which can cover the whole visible range, makes them suitable for light emission in the short to long wavelength part of the visible spectrum [1,2,3]. InGaN quantum well (QW) structures, when used as active regions, play an important role in the high luminescence performance of devices [4,5,6,7]. However, the large lattice mismatch between GaN and InN (10.7% in the *a* direction and 15% in the *c* direction [8]) limits the miscibility of the InGaN alloy. The stress accumulation increases with the increase in the In component in the InGaN QWs, and it also leads to a decrease in thermal stability. Therefore, in the actual growth process of InGaN/GaN MQWs through metal–organic chemical vapor deposition (MOCVD), it is necessary to lower the InGaN growth temperature to typically below 800 °C [9] and maintain a nitrogen (N_2_) atmosphere to improve the incorporation efficiency of In atoms. The presence of hydrogen (H_2_) in the growth environment is usually forbidden because an abundance of hydrogen can lead to the desorption of indium [10]. However, in the subsequent growth process of the p-type GaN layer, a higher temperature and hydrogen atmosphere are needed to activate Mg acceptors [11], which may cause a high thermal budget [12] and lead to a decomposition of the InGaN QW layers [13]. Thus, in order to obtain homogeneous InGaN MQWs and excellent device performance, a study of how hydrogen treatment affects the InGaN/GaN MQW structure and its luminescence property is necessary.

Several articles have reported the effects of hydrogen on InGaN quantum wells [14,15,16,17,18]. Except for the chance of possible damage to the MQW structure, in some cases, H_2_ can also play a role in eliminating In clusters, and thus, inhibiting the thermal decomposition of MQWs [19,20]. Moon et al. [21,22] reported that the use of hydrogen during the growth interruption can effectively suppress the formation of indium-segregated regions in the InGaN well layers and improve the photoluminescence intensity. Suihkonen et al. [9] also used the method of growth interruption and found that the smooth surface morphology of the MQW structure can be obtained with hydrogen treatment. Although there has been a lot of research on the effect of hydrogen, most studies are based on a growth process of MQWs using an atmosphere containing hydrogen, and few studies have been conducted on the effect of using hydrogen in the subsequent growth process of the p-type layer on MQWs, which is important to study for improving the performance of LED devices.

In this paper, we investigate the luminescence characteristics and surface topography of InGaN/GaN MQWs in the presence of hydrogen by simulating the H_2_ growth atmosphere of the p-type cladding layer, which works on the as-grown MQWs directly. Additionally, it is found that exposure to the hydrogen atmosphere leads to the decomposition of the InGaN QWs, as well as to the formation of inhomogeneous In clusters and the degradation of the luminescence property. The experimental results show that a GaN cap layer with an appropriate thickness growing over the MQWs can effectively maintain the integrity of the MQW structure during the hydrogen treatment process and improve the MQW luminescence intensity at room temperature, which may be even higher in comparison to the as-grown sample. The evolution of the surface morphology is also studied using an atomic force microscope (AFM).

## 2. Experimental Procedure

Three InGaN/GaN MQW samples, named A, N1, and H1, were grown on c-plane sapphire substrates through metal–organic chemical vapor deposition (MOCVD). Trimethylgallium (TMGa), trimethylindium (TMIn), and ammonia (NH3) were used as precursors in the epitaxial growth process to provide Ga, In, and N sources, respectively. All the samples were prepared with a two-step growth technology [23,24], in which a GaN buffer layer was grown at a relatively low temperature on the c-plane sapphire substrate firstly; then, this was followed by an unintentionally doped-GaN (u-GaN) epilayer when the growth temperature was raised to 1020 °C. Additionally, a two-period InGaN/GaN MQW was deposited as the active region under the temperature of 750 °C. The growth time for the QW and QB was 150 s and 1100 s, respectively. Moreover, N_2_ acted as the carrier gas for the growth of InGaN and GaN epitaxial layers in all of the above-mentioned preparation processes. The above growth conditions of the three samples were the same. Then, different heat treatments were applied to them. As shown in Table 1, sample A is the as-grown structure without any subsequent heat treatment. For sample N1 and sample H1, a N_2_ and H_2_ atmosphere were introduced during the heat treatment process, respectively. The treatment time and temperature were 230 s at 1020 °C and then 120 s at 950 °C. Such a two-temperature process was set up to simulate the growth condition of the p-type cladding layer and ohmic-contact layer grown during the fabrication of the GaN-based LED structure. In addition, in order to check the protective effect of the GaN cap layer on the MQW structure during the hydrogen treatment, two other samples named H2 and H3 were prepared in which a GaN cap layer was grown on the top of the upmost QB layer of MQWs at 750 °C. The growth time of the GaN cap layer was 1100 s for sample H2 and 3300 s for sample H3. Here, we assumed that the thickness of the GaN layer is approximately proportional to the growth time. It indicates that the cap layer thickness of sample H3 is three times thicker than sample H2. Subsequent hydrogen treatment conditions for H2 and H3 were the same as used for sample H1. The specific growth and heat treatment conditions are shown in Table 1.

High-resolution X-ray diffraction (HRXRD) was performed to measure the indium content and thickness of the InGaN/GaN MQW structure. Temperature-dependent photoluminescence (TDPL) spectra and room-temperature photoluminescence (RTPL) spectra were measured using a 325 nm He-Cd laser in a closed-cycle helium refrigerator from CTI-Cryogenics. Meanwhile, microscopic photoluminescence (u-PL) with a high spatial resolution was performed by using a Nikon A1 confocal optical system excited with a 405 nm laser. The morphology of the samples was measured via an atomic force microscope (AFM).

## 3. Results and Discussion

**Figure 1:**
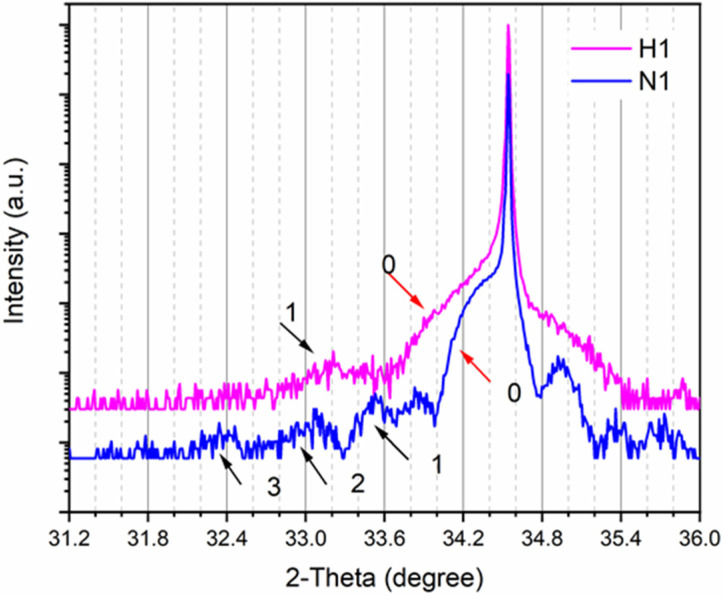
High-resolution X-ray diffraction (HRXRD) ω-2θ scanning curves on (0002) of sample N1 (annealed in N_2_ atmosphere, blue curve) and sample H1 (annealed in H_2_ atmosphere, pink curve). The sharpest peaks at ~34.5° come from GaN diffraction. The arrows indicate the position of satellite peaks.

Figure 1 shows the HRXRD ω-2θ scanning curves of samples N1 and H1, which are heat-treated in a N_2_ and H_2_ atmosphere, respectively. By fitting the experimental data with a global fit program, we can obtain the structural parameters of sample N1 and sample A as shown in Table 2. Since the growth conditions of the MQWs are exactly the same, the variation of structural parameters of sample A and sample N1 is caused by the heat treatment. The specific structural parameters of sample H1 cannot be obtained by fitting the XRD scanning curve due to the poor agreement with the simulation results (not shown in the figure). Additionally, the related data in this line are empty (n.a.) in Table 1. The fitting results indicate that the periodic structure of the MQW has been damaged, which can also be seen from the XRD scanning curves. The third-order satellite peak can be distinguished clearly in sample N1, whereas in hydrogen-treated sample H1, only the first-order satellite peak can be seen in Figure 1. The change in the crystal structure may be due to the etching of the InGaN QW layers by hydrogen, which penetrates through the uppermost GaN QB layer to the first QW layer and gradually penetrates inward. When hydrogen is introduced into the chamber, it may react with the In-rich regions [10,19] and increase the diffusion length of the In atom in InGaN, which causes In atoms in the QW layer to diffuse to the QB layer through the interface, resulting in interface fluctuations and even a partial decomposition of the MQW structure. Meanwhile, the In atoms diffused to the surface have a tendency to escape from the film surface into the chamber, causing a decrease in In content in InGaN.

**Figure 2:**
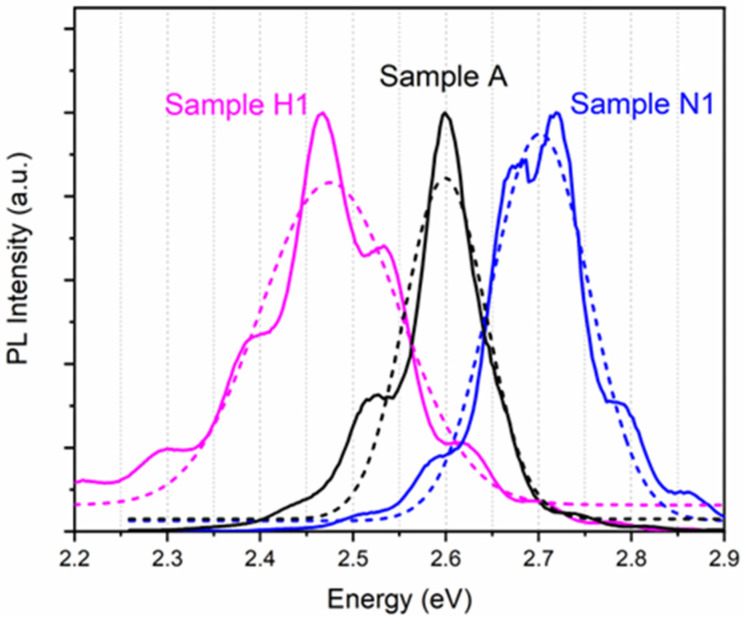
The PL spectra (solid lines) of samples A (black), N1 (blue), and H1 (pink) measured at 30 K. The dashed lines show the Gaussian fitting curves to the measured PL spectra for each sample. The fringes in the spectra are caused by the Fabry–Perot interference effect.

Figure 2 shows the PL spectra (solid lines) of samples A, N1, and H1 measured at 30 K. The additional fringes in the spectra are caused by the Fabry–Perot interference effect. Gaussian fitting is performed on the luminescence spectra, as shown by the dashed lines in Figure 2. Additionally, the peak energy of samples A, N1, and H1 can be obtained as 2.60 eV, 2.70 eV, and 2.48 eV, respectively. The FWHM can also be obtained as 0.10 eV, 0.12 eV, and 0.18 eV for samples A, N1, and H1, respectively. Compared with the as-grown sample A, the decrease in the In content in InGaN QWs is the main reason for the blue shift of the luminescence peak of sample N1. It is obvious that in comparison to samples A and N1, an obvious spectral broadening and a red shift of the MQW luminescence peak happened in the hydrogen-treated sample H1. According to the results of above XRD structural analysis, the In content of InGaN in sample H1 did not increase after the hydrogen treatment. Therefore, we assume that the red shift of the luminescence peak is due to the In aggregation effect [13]. It has been reported that the self-assembled quantum dot-like indium-rich regions can behave as luminescent centers in InGaN [22]. During the H_2_ treatment, it is possible for In atoms to escape from their lattice site and move closer together, forming In clusters [25], which may act as In-rich localized states in InGaN and lead to a red shift of the luminescence peak. The interface roughness of the MQWs and the inhomogeneity of the In clusters may also lead to the increase in the FWHM of sample H1.

**Figure 3:**
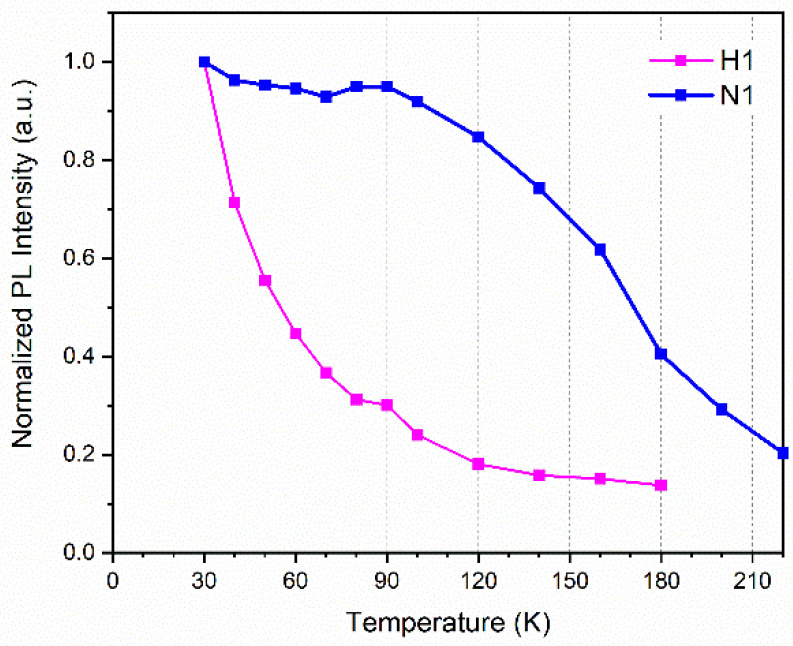
Normalized PL integral intensity as a function of temperature, ranging from 30 K to 220 K. The intensity of sample H1 is only displayed below 180 K because the luminescence intensity at higher temperatures reduces too much to become almost indistinguishable.

**Figure 4:**
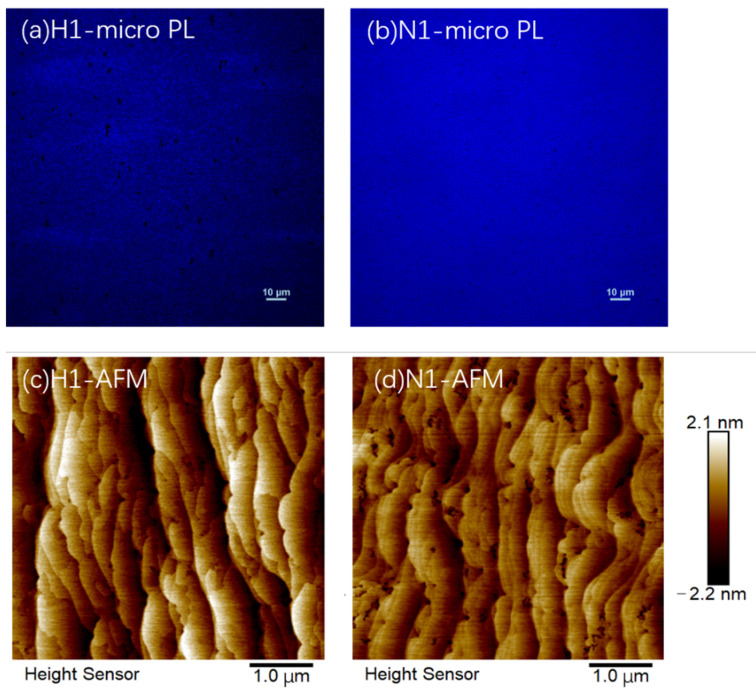
The micro-PL images of (**a**) sample H1 and (**b**) sample N1, where the brightness of dots is proportional to the PL luminescence intensity at each spot; the surface morphology measured by atomic force microscope in tapping mode for sample H1 (**c**), sample N1 (**d**).

To further investigate the hydrogen heat treatment on the optical and structural properties of InGaN MQWs, temperature-dependent PL (TDPL) spectra of the two samples N1 and H1 were measured and the variations in the PL integral intensity with temperature are shown in Figure 3. The luminescence intensity of hydrogen-treated sample H1 declines more rapidly as the temperature increases as compared with sample N1. The near-band-edge luminescence peak becomes very weak, becoming almost indistinguishable when the temperature rises beyond 140 K. This indicates that a large number of defects and dislocations are generated in the hydrogen-treated InGaN QW layers as nonradiative recombination centers. When the temperature increases, the carriers are easily captured by the defects and the nonradiative recombination centers are activated. Therefore, the PL intensity of sample H1 decreases rapidly with the increase in temperature. In order to further study the luminescence properties of the samples, the micro-PL images which have a high spatial resolution of samples H1 and N1 were measured and shown in Figure 4a,b. Both samples were measured with the same excitation power of 110 mW. The brightness of sample H1 is, in total, lower than sample N1, indicating that the overall luminescence intensity of the hydrogen-treated sample is lower. The black dots in the figures are nonradiative recombination centers. It is clearly shown that there are many more nonluminescence dark areas in sample H1 than in sample N1, indicating that the hydrogen treatment may partly damage the MQW structure.

The morphology of samples H1 and N1 is shown in Figure 4c,d, respectively. Both samples show obvious step-bunching-like morphology. The square mean root roughness (Rq) of samples H1 and N1 is 0.837 nm and 0.569 nm, respectively. In addition, the height and width of the surface steps in sample H1 are larger than in sample N1, which indicates that the step bunching phenomenon of the InGaN MQWs will be aggravated after treatment with hydrogen. It has been reported that one of the conditions for step bunching is that the diffusion length of atoms is long enough to expand multiple steps [26]. The hydrogen treatment can increase the diffusion length of In atoms, thus enhancing the step bunching phenomenon. In addition, the above-described XRD and PL results show that more defects and inhomogeneous In clusters are generated in the InGaN QWs after H_2_ treatment. The resulting stress accumulation is considered to be the main reason for the increase in the surface roughness of sample H1. We can also see from Figure 4c,d that there are many more black dots in sample N1, which are related to V-pits. It is known that V-defects are formed at the ends of threading dislocations (TDs) [27,28,29] due to a strain-induced mechanism and the anisotropy of the growth rate. Additionally, V-defects always have In-rich inclusions located in the center of them [30,31]. During heat treatment, In atoms of InGaN/GaN MQWs have a tendency to break free from the lattice, diffuse toward the surface, and nucleate at the end of the TDs. Therefore, it is likely that sample N1 will form more V-defects on the surface. However, the hydrogen treatment can increase the mobility of In atoms and remove the In-rich clusters from the sample surface, which may reduce the density of V-defects on the surface of sample H1.

In the above results, the influence of H_2_ on the internal structure and surface morphology of InGaN/GaN MQWs were studied where the MQW structure was directly exposed to the hydrogen atmosphere. Now, we consider what can we do to decrease the decomposition of InGaN QWs if the hydrogen atmosphere is used in the practical device growth process. Could we even effectively use the hydrogen atmosphere to improve the luminescence property of MQW samples? We speculate that a thicker GaN cap layer grown on the top of the MQW layers may have two effects. The first effect is to reduce the diffusion of hydrogen into the sample; thus, only relatively less hydrogen can penetrate into the InGaN QWs. The second one is to reduce the diffusion of In atoms to the surface, which may improve the surface morphology. In the next several sections, we study two additional hydrogen-treated samples H2 and H3, which have a thicker GaN cap layer grown on the surface, to investigate their luminescence properties and morphology.

**Figure 5:**
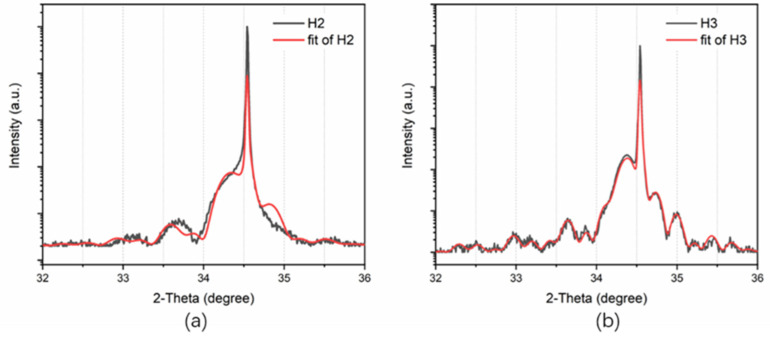
High resolution X-ray diffraction (HRXRD) ω-2θ scanning curves on (0002) of (**a**) sample H2 and (**b**) sample H3. The black and red lines are the measurement data and fitting data, respectively.

**Figure 6:**
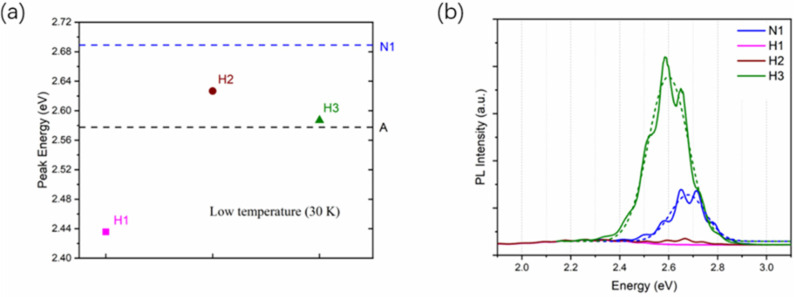
(**a**) PL peak energy distribution of samples treated with hydrogen at 30 K. The dashed lines indicate the peak energy position of sample A and N1 as a reference; (**b**) PL spectra of samples measured at room temperature for samples N1 (blue), H1 (pink), H2 (brown), and H3 (green). The dashed lines show the Gaussian fitting curves of sample H3 and sample N1.

The structural parameters of samples H2 and H3 were obtained by HRXRD measurement, as shown in Table 2. It is worth noting that the thicknesses of the GaN cap layers were taken into account when fitting the XRD results. Therefore, the variations of the parameters of QWs between these samples after heat treatment, as shown in Table 2, are mainly caused by different treatment conditions instead of the GaN cap layers. Figure 5 shows the HRXRD ω-2θ scanning curves of the two samples with a GaN cap layer. The black and red lines are the measurement and simulation data, respectively. It is obvious that the satellite peaks of sample H3 are clearer and its experimental curve is in better agreement with the calculated one, which indicates that sample H3 has a better periodic MQW structure compared with sample H2. Figure 6a shows the PL peak energy of three hydrogen-treated samples compared with samples A and N1 measured at 30 K. The reason why samples A and N1 luminesce at different wavelengths is that the high temperature treatment causes the loss of indium from the QWs. As shown in Table 2, the In content of the QW in sample A is 11.28%, whereas it decreases to 5.60% in sample N1. The decrease in the In content in the QWs in sample N1 causes the blue shift of the luminescence wavelength. It is clearly seen that luminescence peaks in samples H2 and H3 show an obvious red shift with the increase in the cap layer thickness, which is due to the higher In content of the QWs in sample H3, according to the XRD results shown in Table 2. The peak position of sample H2 and H3 gradually approaches the peak energy of the as-grown sample A. Compared with sample H1, the samples with cap layers (H2 and H3) show better thermal stability of the structure and have better interface quality. The In content of the InGaN QW increases from 5.6% in sample N1 to 10.9% in sample H2, indicating that the cap layer can not only reduce the effects of the hydrogen atmosphere effectively, but also can alleviate the influence of high temperature on the structure parameters of the MQWs. It suppresses the decomposition of the InGaN layer and the diffusion of In atoms to the QB layers. As the thickness of the cap layer further increases in sample H3, the QW thickness and In content measured after the heat treatment are basically the same as the as-grown sample A, showing that the cap layer plays a significant role in protecting the InGaN/GaN structure and a thicker GaN cap layer performs better. It was noted that the luminescence wavelength in sample H3 is a little shorter than in sample A although it has a larger In content. This is because the QW width of sample H3 is narrower and the quantum confinement effect [32] is much stronger, leading to the blue shift of the luminescence wavelength.

To further investigate the luminescence efficiency of samples with different cap layer thicknesses, room-temperature photoluminescence spectra were measured under the same laser power excitation and measurement conditions, as shown in Figure 6b. The dashed lines show the Gaussian fitting curves after removing the Fabry–Perot oscillations in the spectra of sample H3 and sample N1. Compared with the as-grown sample N1, hydrogen-treated sample H1 shows no obvious luminescence peak at room temperature. Sample H2 shows a weak luminescence peak, but it is still much weaker than sample N1. This indicates that although the luminescence efficiency of the quantum well is slightly improved after the adoption of a thinner GaN cap layer, it still does not resist the penetration of hydrogen well. It was found that the luminescence intensity was significantly increased by tripling the thickness of the cap layer. The PL peak of H3 even exceeds that of the nitrogen-treated sample N1. This indicates that the interface quality of sample H3 may be remarkably improved, which can effectively improve the photoluminescence intensity of MQWs. It is due to the reduction in hydrogen diffusion into the InGaN QW layers caused by the thicker cap layer. Meanwhile, considering that InGaN may decompose at over 600 °C, it can be concluded that the thicker cap layer also protects the InGaN QW structure from the damage due to the high temperature treatment. 

**Figure 7:**
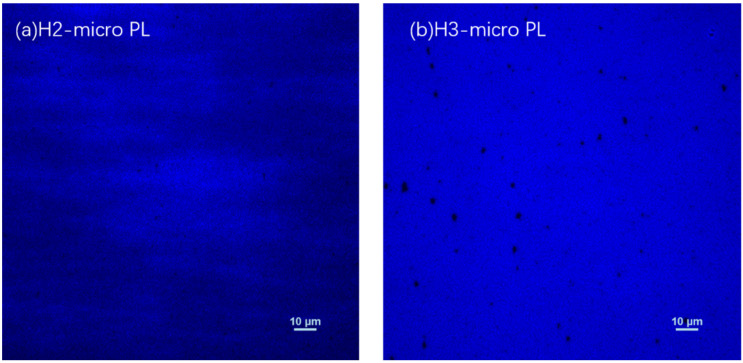
The micro-PL images of (**a**) sample H2 and (**b**) sample H3. The brightness of dots is proportional to the PL luminescence intensity at each spot.

Micro-PL images were measured at room temperature to investigate the uniformity of luminescence, as shown in Figure 7. The excitation power is 110 mW for the two samples, and the detector gains of samples H2 and H3 are 13 and 8 for measurement. The larger overall brightness of Figure 7b, despite being under a lower detector gain, indicates that the luminescence intensity of sample H3 is significantly higher than that of sample H2, which is consistent with the room-temperature PL results. It was also found that there are many more nonradiative dark areas in the PL image of sample H3, suggesting that although the overall luminescence intensity is improved due to the improvement of interface quality, many nonradiative recombination centers are generated in sample H3. This may be due to the fact that the blocking effect of the thick cap layer reduces the elimination effect of In aggregation in the InGaN QWs [21,22] during the heat treatment, which may lead to the formation of nonradiative recombination centers.

**Figure 8:**
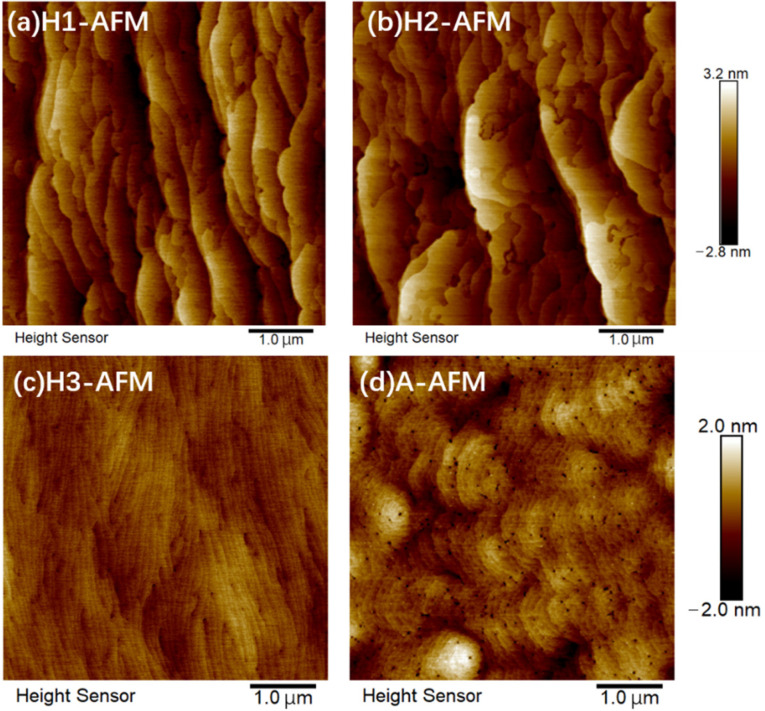
Surface morphology measured by atomic force microscope in tapping mode for samples H1 (**a**), H2 (**b**), H3 (**c**), and reference sample A (**d**).

Figure 8a–c show the surface morphology of three samples treated with hydrogen and Figure 8d shows the morphology of the as-grown sample A as a reference. The scan sizes are 5 × 5 μm^2^ and the scan depth is −2.8 nm to +3.2 nm for samples H1 and H2 and −2.0 nm to +2.0 nm for samples H3 and A. The (100) plane is parallel to the bottom lines of the images. It is worth noting that all surfaces we measured here are GaN surfaces. The square mean root roughness (Rq) of samples H1, H2, H3, and A are 0.837 nm, 0.904 nm, 0.283 nm, and 0.499 nm, respectively. Compared with sample H1, the surface of sample H2 shows a larger step width and depth. As we discussed above, the stress accumulation increases the surface roughness. Despite the fact that a GaN cap layer is adopted to protect InGaN QWs from decomposition in sample H2, the MQW structure is still damaged and the defects and dislocations are produced. A thick GaN cap layer in sample H2 would make the stress accumulation more serious; therefore, the roughness of sample H2 is even larger than that of sample H1. It also indicates that the GaN cap layer of sample H2 cannot resist the penetration of hydrogen well. An obvious morphologic transformation from step bunching to shallower steps [33] has occurred when a GaN cap layer that is three times thicker is adopted in sample H3. The surface morphology of the reference sample A was measured to prove that the surface of samples without or before hydrogen heat treatment would not show the step bunching morphology, as shown in Figure 8d. The maintenance of the shallow step morphology in sample H3 suggests that the cap layer of sample H3 can effectively block the diffusion of hydrogen into the InGaN QWs and obtain a smooth surface with a lower roughness and fewer V-pit defects. The smooth surface also indirectly indicates that the interface quality of sample H3 was improved.

## 4. Conclusions

In this work, the effect of the hydrogen heat treatment on the luminescence and morphology of MOCVD-grown InGaN/GaN MQWs were studied by simulating the growth conditions of p-type cladding layers in the LED device structure. It was found that the hydrogen heat treatment may lead to the decomposition of InGaN QWs and the formation of inhomogeneous In-rich clusters, which act as radiative recombination centers and cause a red shift of the luminescence peak. The defects and stress accumulation generated in the film lead to the degradation of the luminescence efficiency and an increase in surface roughness. It was also found that the hydrogen treatment can effectively reduce the number of V-defects on the surface. By growing a GaN cap layer with an appropriate thickness on MQWs, the luminescence efficiency of samples after hydrogen heat treatment can be improved strongly, indicating that the blocking effect of the cap layer on hydrogen diffusion and high temperature can effectively maintain the integrity of the MQW structure. Additionally, a morphologic transformation from step bunching to shallower steps can be obtained in the sample with a thicker cap layer.

## Figures and Tables

**Table 1:** Growth and heat treatment conditions of samples.

Samples	Growth Time of QW/QB (s)	Growth Time of Cap Layer (s)	Treatment Atmosphere	Treatment Conditions
A	150/1100	0	none	none
N1	150/1100	0	N_2_	230 s at 1020 °C and 120 s at 950 °C
H1	150/1100	0	H_2_	230 s at 1020 °C and 120 s at 950 °C
H2	150/1100	1100	H_2_	230 s at 1020 °C and 120 s at 950 °C
H3	150/1100	3300	H_2_	230 s at 1020 °C and 120 s at 950 °C

**Table 2:** Structural parameters of samples determined by HRXRD measurements.

Samples	Thickness of QW (nm)	In Content of QW	Thickness of QB (nm)	In Content of QB
A	2.69	11.28%	12.58	1.88%
N1	4.70	5.60%	8.70	1.56%
H1	n.a.	n.a.	n.a.	n.a.
H2	3.51	10.90%	11.21	1.17%
H3	2.40	12.60%	12.10	1.65%

## Data Availability

The datasets used and/or analyzed during the current study are available from the corresponding author on reasonable request. The data that support the findings of this study are available upon reasonable request from the authors.
